# Prevalence, predictors, and management for balloon uncrossable or undilatable lesions in patients undergoing percutaneous coronary intervention with in-stent restenosis chronic total occlusion

**DOI:** 10.3389/fcvm.2023.1095960

**Published:** 2023-05-30

**Authors:** Yong Wang, Ai-jie Hou, Bo Luan, Xiao-jiao Zhang, Zhao-yu Li, Xiao-yang Pei

**Affiliations:** ^1^ Department of Cardiology, Shenzhen Luohu Hospital Group Luohu People’s Hospital (The Third Affiliated Hospital of Shenzhen University), Shenzhen, China; ^2^ Department of Cardiology, The People’s Hospital of China Medical University, The People’s Hospital of Liaoning Province, Shenyang, China; ^3^ Department of Cardiology, Second Affiliated Hospital Zhejiang University School of Medicine, Hangzhou, China

**Keywords:** chronic total occlusion, balloon uncrossable or undilatable, in-stent restenosis, percutaneous coronary intervention, management

## Abstract

**Background:**

Percutaneous coronary intervention for in-stent restenosis (ISR) chronic total occlusion (CTO) has been a great challenge. There are occasions when the balloon is uncrossable or undilatable (BUs) even though the guidewire has passed, leading to failure of the procedure. Few studies have focused on the incidence, predictors, and management of BUs during ISR-CTO intervention.

**Methods:**

Patients with ISR-CTO were recruited consecutively between January 2017 and January 2022 and divided into two groups based on the presence of BUs. The clinical data of the two groups (BUs group and non-BUs group) were retrospectively analyzed and compared to explore the predictors and clinical management strategies of BUs.

**Results:**

A total of 218 patients with ISR-CTO were included in this study, 23.9% (52/218) of whom had BUs. The percentage of ostial stents, stent length, CTO length, the presence of proximal cap ambiguity, moderate to severe calcification, moderate to severe tortuosity, and J-CTO score were higher in the BUs group than in the non-BUs group (*p* < 0.05). The technical success rate and the procedural success rate were lower in the BUs group than in the non-BUs group (*p* < 0.05). Multivariable logistic regression analysis showed that ostial stents (OR: 2.011, 95% CI: 1.112–3.921, *p* = 0.031), the presence of moderate to severe calcification (OR: 3.383, 95% CI: 1.628–5.921, *p* = 0.024) and moderate to severe tortuosity (OR: 4.816, 95% CI: 2.038–7.772, *p* = 0.033) were independent predictors of BUs.

**Conclusion:**

The initial rate of BUs in ISR-CTO was 23.9%. Ostial stents, presence of moderate to severe calcification, and moderate to severe tortuosity were independent predictors of BUs.

## 1. Introduction

Chronic total occlusion (CTO) is one of the most challenging and complex conditions in interventional cardiology. Previous studies have demonstrated that CTO accounts for approximately 16%–18% of patients with coronary artery disease (CAD) who underwent a coronary angiography (1, 2). It has been reported that in-stent restenosis (ISR) CTO, a subset of CTO lesions, makes up 5%–25% of all cases of CTO lesions (3, 4). Compared with *de novo* CTO, interventions in ISR-CTO have a lower success rate (4, 5), thus making it the most complex and challenging subset. Recent advancements in the surgical technique and equipment, especially the development and application of retrograde techniques and hybrid strategies, have led to a significant improvement in the success rate of ISR-CTO intervention. Recent studies reported that there were comparable success rate in *de novo* CTO and ISR-CTO (6, 7). Nevertheless, ISR-CTO intervention remains more challenging due to the presence of in-stent calcification, heterogeneous in-stent neoatherosclerosis, stent under-expansion, multilayer stents, previous stented vascular tortuosity, and ostial stents.

The primary cause for the failure of CTO PCI was the failure of guidewire passing through the CTO lesion (8). Even when the guidewire has been passed through successfully, balloon undilatable CTOs (BUs) may also contribute to procedure failure. Previous studies reported that the general incidence of BUs in CTO PCI could be 8.5%–12% (9, 10), but the incidence during ISR-CTO interventions remains unknown. Furthermore, the predictors and optimal management strategies for BUs during ISR-CTO intervention also remain unclear. This study aimed to investigate the incidence of BUs after successful guidewire passing through an ISR-CTO lesion, explore the influencing factors, and summarize the clinical management strategies. The goal of this study was to provide a reference for optimizing the management of ISR-CTO.

## 2. Methods

### 2.1. Study population

The study consecutively enrolled 218 patients with ISR-CTO in the People's Hospital of Liaoning Province from January 2017 to January 2022. The exclusion criteria were patients with NYHA class IV cardiac function and patients who did not agree to be enrolled. The medical records and coronary angiographic characteristics of the patients were recorded in detail so as to clearly analyze the characteristics of the CTO lesions. The procedural strategy for each case was discussed before PCI and carefully performed to maximize the success rate of the procedure in ISR-CTO.

### 2.2. Definitions

CTO was defined as an occlusion with a thrombolysis in myocardial infarction (TIMI) grade 0 flow for more than three months, documented angiographically or described clinically (11). BUs was defined as a failure in balloon advancement within the target lesion after successfully guidewire crossing into true lumen distal to the occlusion, or the failure to adequately expanding of the CTO lesion despite the application of different types of 1:1 balloon under the maximum dilating pressure of 20 atm (10). ISR-CTO was defined as a CTO lesion located in stent or within 5 mm proximal or distal to the stent (12). CTO scores were used to predict the possibility of passing the guidewire through the CTO lesion with 30 min to assess the difficulty of the procedure (13). Technical success was defined as a successful stent implantation with <30% residual stenosis and a distal TIMI grade 3 flow. Procedural success was defined as a technical success with no severe complications. Severe complications in this study defined as coronary perforation, stent entrapment, cardiac arrest, and cardiac tamponade requiring pericardiocentesis.

### 2.3. Interventional procedures

The radial artery was the preferred access site for all the individuals, while the right radial artery and the right femoral artery were preferred when a bilateral angiography was required. In some specific patients, the access was determined by the operators. Interventional procedures were first attempted with the antegrade technique (antegrade wire escalation, knuckle technique, and parallel-wire technique, et al). If the antegrade attempt was failed, the retrograde technique would be adopted (retrograde wire escalation, CART technique, and Reverse CART technique). The intraluminal imaging examinations was determined by the operators. The perioperative antithrombotic treatment strategies for the patients was developed by cardiology specialists. The observational study was approved by the ethics committee of the People's Hospital of Liaoning Province and written informed consent was obtained from all patients.

### 2.4. Statistical analysis

Statistical analysis was performed using SPSS version 20.0 (IBM, USA). Normally distributed continuous variables were presented as the mean ± standard deviation, and differences between the two groups were compared using Student's *t*-test. Medians were used to represent data that were not normally distributed, and the Mann-Whitney *U* test was used for data comparisons between two groups. Categorical data were presented in the form of rates or percentages, and the chi-square test or Fisher's exact test were performed for comparisons between the two groups. Univariable regression was performed to analyze the factors associated with BUs and multivariable logistic regression analysis was performed to determine the predictors of BUs. All tests were two-sided, and *p*-values < 0.05 were considered significant.

## 3. Results

### 3.1. Baseline characteristics

A total of 218 ISR-CTO individuals were included in this study, with BUs accounting for 23.9% (52/218). Baseline characteristics were shown in Table 1. There were no differences between BUs and non-BUs patients with regards to age, gender, current smoker diabetes mellitus, hypertension, dyslipidemia, stroke, previous myocardial infarction (MI), previous history of CAD, Peripheral vascular disease, previous heart failure (*p* > 0.05). However, patients with BUs had a higher prevalence of prior CAGB (23.1% vs 8.4%, *p* = 0.012) (Table 1).

**Table 1:** Clinical characteristics of study population.

Variables	non-BUs group (*n* = 166)	BUs group (*n* = 52)	*p*-value
Age (years)	65.1 ± 9.4	65.1 ± 8.3	0.979
Gender (female), *n* (%)	65 (39.2%)	19 (36.5%)	0.870
Current smoker, *n* (%)	95 (57.2%)	31 (59.6%)	0.872
Diabetes Mellitus, *n* (%)	65 (39.2%)	22 (42.3%)	0.746
Hypertension, *n* (%)	110 (66.3%)	38 (73.1%)	0.399
Dyslipidaemia on admission, *n* (%)	105 (63.3%)	35 (67.3%)	0.623
Previous Stroke, *n* (%)	9 (5.4%)	4 (7.7%)	0.515
Previous MI, *n* (%)	53 (31.9%)	19 (36.5%)	0.613
Family history of CAD, *n* (%)	39 (23.5%)	10 (19.2%)	0.573
CABG, *n* (%)	14 (8.4%)	12 (23.1%)	**0**.**012**
Peripheral vascular disease, *n* (%)	19 (11.4%)	8 (15.4%)	0.472
Previous HF, *n* (%)	33 (19.9%)	11 (21.2%)	0.845
LVEF (%)	43.2 ± 5.7	42.5 ± 6.0	0.709
ACS, *n* (%)	42 (25.3%)	14 (26.9%)	0.856

MI, myocardial infarction; CAD, coronary artery disease; HF, heart failure; CABG, coronary artery bypass grafting; LVEF, left ventricular ejection fraction; ACS, acute coronary syndrome.

Bold values means *p* < 0.05.

### 3.2. Angiographic and procedural characteristics of the studied patients

Angiographic and procedural characteristics of the studied patients were shown in Tables 2, 3. No significant differences were observed regarding to time since stent implantation, CTO vessels, tandem occlusions, microchannels, epicardial collaterals, septal collateral, Werner score 0–1, stent diameter, blunt stump (*p* > 0.05). The BUs group had a higher prevalence of ostial stent, proximal cap ambiguity, moderate/severe calcification and moderate/severe tortuosity (*p* < 0.05). Meanwhile, BUs patients had a higher stent length, CTO length and J-CTO score (*p* < 0.05) (Table 2).

**Table 2:** Angiographic characteristics of the studied patients.

Variables	non-BUs group (*n* = 166)	BUs group (*n* = 52)	*p*-value
LAD CTO, *n* (%)	59 (35.5%)	20 (38.5%)	0.806
LCX CTO, *n* (%)	39 (23.5%)	10 (19.2%)	
RCA CTO, *n* (%)	68 (41.0%)	22 (42.3%)	
Tandem occlusions, *n* (%)	38 (22.9%)	12 (23.1%)	1.000
Microchannels, *n* (%)	31 (18.7%)	11 (21.2%)	0.690
Ostial Stent, *n* (%)	26 (15.7%)	15 (28.8%)	**0**.**042**
Epicardial collaterals, *n* (%)	56 (33.7%)	17 (32.7%)	1.000
Septal collaterals, *n* (%)	74 (44.6%)	22 (42.3%)	0.873
Werner score 0–1, *n* (%)	36 (21.7%)	13 (25.0%)	0.704
Stent Diameter (mm)	3.1 ± 0.37	3.0 ± 0.38	0.078
Stent Length (mm)	34.3 ± 8.0	39.4 ± 8.6	<**0**.**001**
CTO length (mm)	19.2 ± 6.1	23.5 ± 7.5	<**0**.**001**
Proximal cap ambiguity, *n* (%)	29 (17.5%)	17 (32.7%)	**0**.**031**
Blunt stump, *n* (%)	29 (25.3%)	16 (30.8%)	0.474
Moderate/severe calcification, *n* (%)	30 (18.1%)	17 (32.7%)	**0**.**033**
Moderate/severe tortuosity, *n* (%)	29 (17.5%)	16 (30.8%)	**0**.**049**
J-CTO score	2.7 ± 0.6	3.0 ± 0.7	**0**.**001**

LAD, left anterior descending artery; CTO, chronic total occlusion; LCX, left circumflex artery; RCA, right coronary artery.

Bold values means *p* < 0.05.

**Table 3:** Procedural characteristics of the studied patients.

Variables	non-BUs group (*n* = 166)	BUs group (*n* = 52)	*p*-value
**Access site**			
Radial, *n* (%)	30 (18.1%)	7 (13.5%)	0.870
Femoral, *n* (%)	8 (4.8%)	3 (5.8%)	
Radial + Femoral, *n* (%)	106 (63.9%)	34 (65.4%)	
Right femoral + left femoral, *n* (%)	22 (13.3%)	8 (15.4%)	
Bilateral injection, *n* (%)	128 (77.1%)	42 (80.8%)	0.702
Initial AWE, *n* (%)	133 (80.1%)	38 (73.1%)	0.334
Initial retrograde, *n* (%)	33 (19.9%)	14 (26.9%)	
**Finally crossing strategies**			
Antegrade	92 (55.4%)	20 (38.5%)	**0**.**039**
Retrograde	74 (44.6%)	32 (61.5%)	
IVUS/OCT used, *n* (%)	119 (71.7%)	42 (80.8%)	0.211
Procedural time (min)	85.8 ± 25.6	118.4 ± 27.4	**0**.**001**
Fluoroscopy time (min)	58.2 ± 18.8	74.1 ± 18.5	<**0**.**001**
Contrast volume, (ml)	217.8 ± 65.5	258.4 ± 76.4	<**0**.**001**
Technical success, *n* (%)	158 (95.2%)	44 (84.6%)	**0**.**027**
Procedural success, *n* (%)	151 (91.0%)	39 (76.9%)	**0**.**014**

AWE, antegrade wire escalation; IVUS, intravscular ultrasound; OCT, optical coherence tomography.

Bold values means *p* < 0.05.

There were no differences between the two groups with regard to access site, bilateral injection, initial AWE, initial retrograde, and intravascular ultrasound (IVUS) used (*p* > 0.05). However, BUs group had a higher rate of retrograde crossing strategy, a longer procedural time and fluoroscopy time, a higher contrast volume (*p* < 0.05).The technical success rate and procedural success rate were significantly lower in BUs than in the non-BUs group (*p* < 0.05) (Table 3).

### 3.3. Complications of the studied patients

One case of vascular access complication was observed in the BUs group (1.9%), while in the non-BUs group the incidence was 1.8%. The BUs group had two cases (3.8%) of donor vessel dissection, while in the non-BUs group had four cases (2.4%). The patients in all donor vessel dissection cases received stent implantations. One case of collateral dissection was observed in the BUs group (1.9%) and four cases (2.4%) were found in the non-BUs group. Additionally, one case (1.9%) of target vessel perforation was observed in the BUs group while one more (1.2%) was found in the non-BUs group. Patients from both cases received emergency coronary artery bypass grafts (CABG). Three cases (5.8%) of collateral perforation were observed in the BUs group while two cases (1.2%) were found in the non-BUs group. Pericardiocentesis to treat pericardial tamponade was performed in four patients (7.7%) in the BUs group and four patients (2.4%) in the non-BUs group. The total incidence of complications was significantly higher in the BUs group than in the non-BUs group (26.9% vs. 12.0%, *p* = 0.015) (Table 4).

**Table 4:** Procedural complications.

Variables	non-BUs group (*n* = 166)	BUs group (*n* = 52)	*p*-value
Vascular access complication, *n* (%)	3 (1.8%)	1 (1.9%)	1.000
Donor vessel dissection, *n* (%)	4 (2.4%)	2 (3.8%)	0.630
Collateral dissection, *n* (%)	4 (2.4%)	1 (1.9%)	1.000
Target vessel perforation, *n* (%)	2 (1.2%)	1 (1.9%)	0.560
Collateral perforation, *n* (%)	2 (1.2%)	3 (5.8%)	0.089
Tamponade requiring pericardiocentesis, *n* (%)	4 (2.4%)	4 (7.7%)	0.095
Emergency CABG, *n* (%)	1 (0.6%)	2 (3.8%)	0.142
Complications in total, *n* (%)	20 (12.0%)	14 (26.9%)	**0**.**015**

CABG, coronary artery bypass grafting.

Bold values means *p* < 0.05.

### 3.4. Predictors of BUs

A univariable regression model was used separately for each of the following covariates: Ostial Stent, Stent Length, CTO length, Moderate/severe calcification, Moderate/severe tortuosity and J-CTO score. Covariates that showed that Ostial Stent, Moderate/severe calcification, Moderate/severe tortuosity were significantly associated BUs. Multivariable logistic regression analysis showed that Ostial Stent (OR: 2.011, 95% CI: 1.112–3.921, *p* = 0.031), Moderate/severe calcification (OR: 3.383, 95% CI: 1.628–5.921, *p* = 0.024), Moderate/severe tortuosity (OR: 4.816, 95% CI: 2.038–7.772, *p* = 0.033) were independent predictors of BUs (Table 5).

**Table 5:** Univariate and stepwise multivariate logistic regression analysis of BUs.

	Univariate analysis			Multivariate analysis		
	OR	95% CI	*P* value	OR	95% CI	*p* value
Ostial Stent, *n* (%)	2.120	1.121–3.681	**0**.**026**	2.011	1.112–3.921	**0**.**031**
Stent Length (mm)	1.152	0.921–2.920	0.127	–	–	–
CTO length (mm)	1.237	0.781–1.823	0.628	–	–	–
Moderate/severe calcification, *n* (%)	3.214	1.612–5.587	**0**.**021**	3.383	1.628–5.921	**0**.**024**
Moderate/severe tortuosity, *n* (%)	4.192	2.221–7.618	**0**.**030**	4.816	2.038–7.772	**0**.**033**
J-CTO score	1.212	1.021–2.018	0.025	1.301	0.826–1.926	0.165

Bold values means *p* < 0.05.

### 3.5. Clinical management of BUs

**Figure 1:**
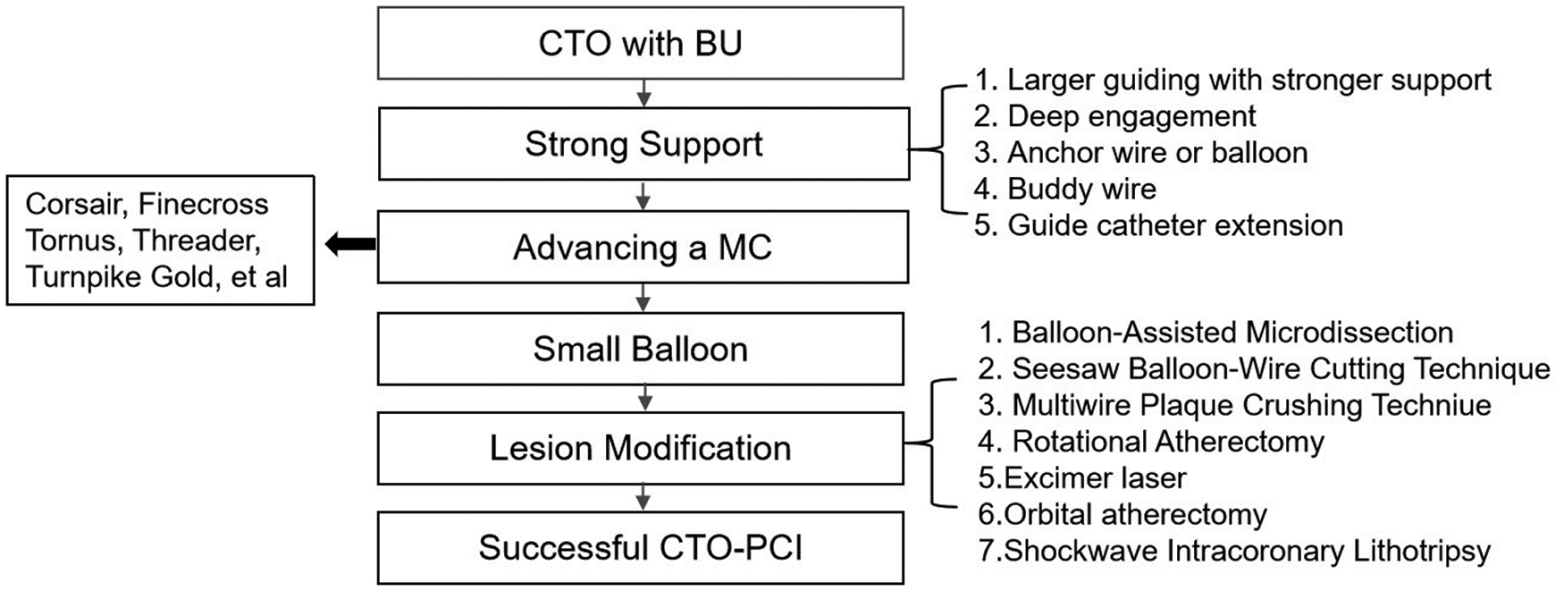
Clinical management of BUs in chronic total occlusion. BU, balloon uncrossable or undilatable; CTO, chronic total occlusion; MC, microcatheter.

Among the 52 patients with BUs, following techniques such as strong guiding and catheter support, anvancement of microcatheters, application of small balloons, and plaque modification (Figure 1), all of the patients received high-pressure balloon inflations (100%); 52% underwent a cutting balloon angioplasty, 38% received a scoring balloon, and 28% went through rotational atherectomy. A final technical success rate of 84.6% (44/52) and a final procedural success rate of 76.9% (39/52) were achieved. For the non-BUs patients, 95.8% (158/166) achieved final technical success and 91.0% (151/166) achieved procedural success. Overall, 92.7% (202/218) of patients with ISR-CTO achieved technical success and 87.2% (190/218) achieved procedural success.

## 4. Discussion

In this study, we found that the incidence of BUs in patients with ISR-CTO was 23.9%. Compared with patients in the non-BUs group, patients with BUs had higher procedural complexity, lower success rates, and a higher incidence of PCI related complications. Ostial stent, moderate to severe calcification, and moderate to severe tortuosity were independent predictors of BUs.

ISR-CTO accounts for approximately 5%–25% of all CTO lesions (3, 4). Compared with *de novo* CTO, ISR-CTO interventions are more challenging. For bare-metal stent CTO, Abbas et al. (4) found that the success rate of PCI was similar to that in *de novo* CTO (63% vs. 70%). Because of the lack of retrograde and hybrid surgical strategies, the success rates of PCI in patients with CTO were generally lower. Although the study stated that the main reason for the failure of the procedure was the failure of the guidewire to pass the CTO lesion, it also found that patients with ISR-CTO had a significantly higher BUs rates than patients with *de novo* CTO (4). Werner et al. found that even with the retrograde technique, the success rate of PCI in patients with ISR-CTO was significantly lower than those in patients with *de novo* CTO (70% vs 85%) (14). With recent advances in techniques and the development of new devices, the success rate of ISR-CTO intervention has increased significantly. Currently, in large medical centers with experienced operators, ISR-CTO intervention has a similar success rate and complication incidence to those in *de novo* CTO interventions (6, 7). In this study, the overall success rate of ISR-CTO interventions was found to be 87.2%. Although a relatively satisfied success rate was achieved, ISR- CTO intervention still faces great challenges.

Although it has been reported in previous studies that the general incidence of BUs in CTO-PCI was 8.5%–12% (9, 10), no study has focused on the analysis of ISR-CTO, a subgroup of CTO. Thus, the incidence of BUs in ISR-CTO interventions remained unknown. In this study, the incidence of BUs in ISR-CTO intervention was found to be 23.9%, which is significantly higher than the previously reported rate of BUs in patients with CTO. This indirectly suggested that ISR-CTO interventions are more challenging, which was consistent with the conclusions of previous studies (4). The causes of BUs can be attributed to three main categories: The first category includes anatomical and lesion characteristics (heavy calcification, vascular tortuosity, and long lesions) prior to stent implantation. The second category were latrogenic (calcification modification unsatisfied, small stent size, and the guidewire passed through the stent mesh). The third category comprises of post-stenting causes like fibrous hyperplasia, calcification, and multilayer stents.

BUs are important factors that lead to PCI failure, but their predictors remain unclear. It was found that patients with previous stent implantations may have longer CTO lesions (15) and more heavily calcified lesions (16). Consequently, calcified lesions would affect balloon passage or dilation. This study found a higher percentage of patients who had undergone a CABG procedure with ISR-CTO in the BUs group. This was because patients who have received a CABG tend to have heavier calcification in the lesion (17) and a more complex anatomy. Also, in this study, it was found that the lesions of patients in the BUs group were longer, more tortuous, and presented a significantly higher proportion of moderate to severe calcification than those of patients in the non-BUs group; tortuosity and the presence of moderate to severe calcification were predictors of BUs. These results suggested that the risk factors associated with BUs in patients with *de novo* CTO also apply to patients with ISR-CTO. Previous studies have reported that a higher proportion of ostial CTO have lesions with proximal-cap ambiguity, moderate to severe calcification, and a longer shape compared to lesions in non-ostial CTO. Long and calcified lesions resulted in a higher chance of BUs, making the procedural more complex (18). Consistent with previous studies, we found a higher proportion of patients with ostial CTO in the BUs group and that ostial CTO was a predictor of BUs. Different from *de novo* BUs, post-stenting heterogeneous fibrous hyperplasia, calcification, and multilayer stent structure in patients with ISR-CTO increased the procedural difficulty. Moreover, consistent with previous studies, this study also found that BUs can lead to an increased procedural risk and decreased success rate (9, 10). This is presumably due to the higher J-CTO scores and more complex anatomy in patients in the BUs group.

It is necessary to ensure that the balloon is adequately expanding in patients with BUs prior to stenting. Otherwise, the stent may fail to pass or expanded unsatisfied, which in turn cause in-stent thrombosis and ISR (19). Meanwhile the management of BUs is also quite important (Figure 1). Firstly, it is important to ensure adequate support of the guide catheter. When guiding support is inadequate, a Guidezilla extension catheter should be used for support augmentation. A multiple guidewire technique and balloon anchoring technique are other options for stronger support. A corsair microcatheter with a micro-expansion function is preferable to improve the success rate of the intervention (20). High-pressure balloon inflations are typically used for BUs. A 1:1 sized non-compliant balloon is dilated under high pressure with a median maximum dilation pressure of 25 atm [IQR 20–30]) (21). High-pressure balloon inflations are the simplest and most widely available technique that can be repeated multiple times. However, it carries the risk of balloon rupture or vascular perforation. Since this study focused on ISR cases, high-pressure balloon inflations were used in all recruited patients (100%). The buddy wire technique is also a good option (22). Additionally, a cutting balloon (23) could be used to create controlled incisions in the vessel wall to help the target vessel dilated adequately. Considering the poor pass-through capacity of cutting balloons, it is recommended that the procedure be assisted by the application of a strong support catheter and a Guidezilla or anchoring balloon. In this study, the proportion of patients who underwent cutting balloon angioplasty was 52%. In addition, the proportions of patients who received scoring balloon angioplasty and rotational atherectomy (RA) were 38% and 28%, respectively.

An RA in ISR-CTO is extremely challenging. Studies have confirmed that RA can ablate not only metallic stent struts but also calcification at the base of the stent. Therefore, this technique can be used for lesion modification of ISR-CTO (23, 24). The studies that focused on ISR RAs all had small sample sizes (11–16 cases), reported good treatment outcomes and minimal complications in patients included (25–27). The purpose of RA is to ablate the sites of failed stent expansion and sub-stent calcification, as opposed to stand-alone debulking. Therefore, a smaller burr size should be used. An initial burr-to-artery ratio of 0.5 was used in this study. A small-sized burr was used first, and a balloon with high-pressure inflation was used for dilation after RA. If the result was unsatisfactory, a stepped RA strategy with larger burrs was used. Before RA, it is necessary to ensure that the guidewire is in the distal true lumen and has not passed through the stent mesh. Intravascular ultrasonography can then be performed to further clarify the route of the guidewire. During the RA procedure, it is important to avoid using excessive force and pushing the burr forward too rapidly. Instead, the RA should be performed at a low speed to increase RA efficiency and avoid burr entrapment. Moreover, the RA should be performed multiple times until the burr passes through the lesion. RA procedure for ISR-CTO is quite a high risk procedure and requires a rich experience to avoid burr entrapment. Following the RA procedure, the calcification ring is opened, and the stent struts thinned, thus facilitating the subsequent devices passing and expansion of the high-pressure inflation balloon to achieve better stent expanding. In this study, all 10 patients who underwent RA had no burr entrapment. One case of slow to no recurrent flow was observed, and a TIMI grade 3 flow was later restored. There were no coronary dissection and perforation in these patients. Other techniques, including laser (28), Shockwave intracoronary lithotripsy (29), and orbital atherectomy (30), could be used for treating ISR. However, these devices are not available in our hospital.

This study had some limitations. Firstly, this was a single-center study with a small sample size, which can lead to selective bias. Secondly, the duration of patient recruitment in this study was six years (from 2017 to 2022). During this period, the procedural success rate gradually increased with the accumulation of procedural experience and advances in the devices used. The success rate of interventions in early patients may be different from that of recent patients, which may have impacted the study results. Thirdly, Core laboratory was not used in this study. That is a limitation of this study. Finally, the patients included in this study were from a specific population of ISR-CTO with high J-CTO scores. Therefore, the findings may not apply to the general population. Future multicenter studies at a larger scale and data obtained from highly experienced centers and operators are needed to validate our conclusions.

## 5. Conclusion

The initial rate of BUs in ISR-CTO was 23.9%. Ostial stents, presence of moderate to severe calcification, and moderate to severe tortuosity were independent predictors of BUs.

## Data Availability

The raw data supporting the conclusions of this article will be made available by the authors, without undue reservation.
